# Telomere-led meiotic chromosome movements: recent update in structure and function

**DOI:** 10.1080/19491034.2020.1769456

**Published:** 2020-06-07

**Authors:** C. Y. Lee, C. G. Bisig, M. N. Conrad, Y. Ditamo, L. Previato de Almeida, M. E. Dresser, R. J. Pezza

**Affiliations:** a Cell Cycle and Cancer Biology Research Program, Oklahoma Medical Research Foundation, Oklahoma City, OK, USA; b Facultad de Ciencias Químicas, Dpto. Química Biológica Ranwel Caputto-CIQUIBIC, Universidad Nacional de Córdoba, Córdoba, Argentina; c Department of Cell Biology, University of Oklahoma Health Science Center, Oklahoma City, OK, USA

**Keywords:** LINC, Mps2, Myo2, Csm4, rapid prophase movements (RPMs)

## Abstract

In S. cerevisiae prophase meiotic chromosomes move by forces generated in the cytoplasm and transduced to the telomere via a protein complex located in the nuclear membrane. We know that chromosome movements require actin cytoskeleton [13,31] and the proteins Ndj1, Mps3, and Csm4. Until recently, the identity of the protein connecting Ndj1-Mps3 with the cytoskeleton components was missing. It was also not known the identity of a cytoplasmic motor responsible for interacting with the actin cytoskeleton and a protein at the outer nuclear envelope. Our recent work [36] identified Mps2 as the protein connecting Ndj1-Mps3 with cytoskeleton components; Myo2 as the cytoplasmic motor that interacts with Mps2; and Cms4 as a regulator of Mps2 and Myo2 interaction and activities (Figure 1). Below we present a model for how Mps2, Csm4, and Myo2 promote chromosome movements by providing the primary connections joining telomeres to the actin cytoskeleton through the LINC complex.

In meiosis, DNA replication is followed by two rounds of chromosome segregation. In the first round, prophase progresses through distinct stages, during which homologous chromosomes pair (leptonema-zygonema). Synaptonemal complex is then formed, a proteinaceous structure formed in between each pair of homologous chromosomes stabilizing their interaction, promoting synapsis (zygonema-pachynema).

Mutations in genes that regulate pairing and synaptonemal complex dynamics are invariably associated with increased errors in meiotic chromosome segregation. This is particularly relevant considering that chromosome segregation errors occur in about 10%-30% of human germ cells, resulting in aneuploid gametes, ultimately resulting in aneuploid conceptus abortions before term, and a number of aneuploid births with physical disabilities and mental retardation [1–3].

Chromosome interactions (pairing and synapsis) are accompanied by prominent active chromosome movements that are conserved in organisms from yeast to mammals [4–18]. RPMs have been implicated in promoting homologous chromosome pairing and synapsis [16,19–26], reducing non-homologous interactions [6,20,21,27,28], and resolving chromosome interlocks [29,30]. Thus, chromosome movements participate in critical meiotic events required for normal progression of gametogenesis.

In S. cerevisiae prophase meiotic chromosomes move by forces generated in the cytoplasm and transduced to the telomere via a conserved protein complex located in the nuclear membrane. From previous studies, we know that budding yeast chromosome movements require an intact actin cytoskeleton [13,31] and the proteins Ndj1, Mps3, and Csm4 at the nuclear membrane. The telomeric Ndj1 binds the nucleoplasmic domain Mps3, which contains a SUN domain [32–35]. However, until recently, the identity of the protein connecting Ndj1-Mps3 with the machinery that generates the forces at the cytoskeleton was missing. It was also not known the identity of a predicted cytoplasmic motor responsible for interacting with the actin cytoskeleton and the outer nuclear portion of a protein at the outer nuclear envelope. Our recent work [36] identified Mps2 as the protein connecting Ndj1-Mps3 with cytoskeleton components; Myo2 as the cytoplasmic motor that interacts with Mps2; and the Cms4 protein as a possible regulator of Mps2 and Myo2 interaction and activities (Figure 1). Below we discuss a model in which the interaction between Mps2, Csm4, and Myo2 promotes chromosome movements by providing the primary connections joining telomeres to the actin cytoskeleton through the LINC complex.

**Figure 1.**
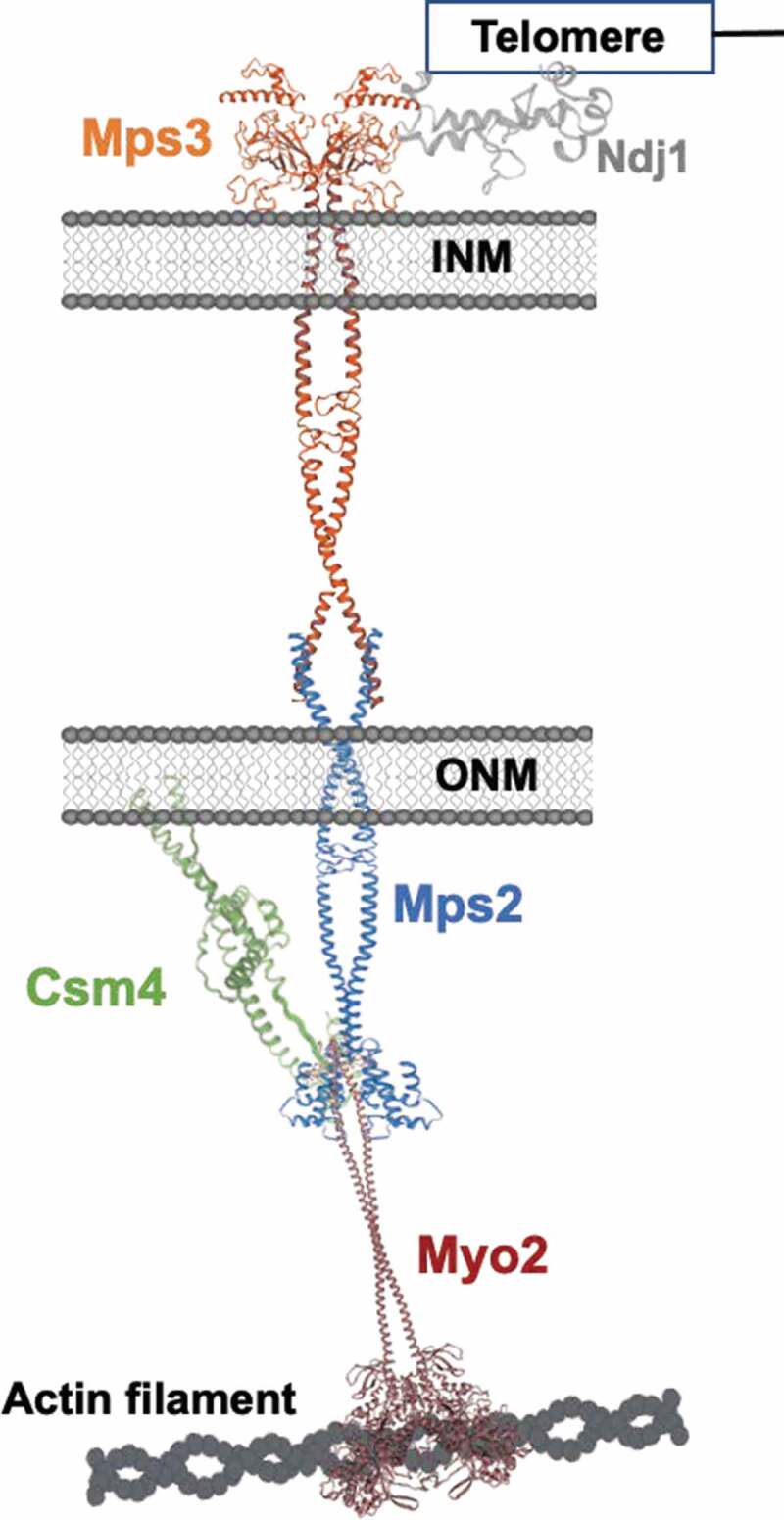
Schematic of a model of the engine generating RPMs in budding yeast. The structure diagram corresponding to the proteins participating in the mechanism was obtained using https://swissmodel.expasy.org and are not shown at scale.

## Mps2 acts as a KASH-like protein connecting the LINC complex to cytoskeleton components

Previous work showed that the meiosis-specific Mps3-Ndj1 complex interacts with telomeres [33]. We then reasoned that known Mps3 interactors may be candidates to identify the missing LINC protein connecting the telomere to the cytoskeleton. Indeed, in mitotic cells Mps3-Mps2 work together in the spindle pole body [37]. Although Mps2 does not show a conserved KASH domain as usually observed in KASH proteins, Mps2 is an integral membrane protein of the nuclear envelope with a transmembrane domain near the C terminus [37–39]. We then tested whether Mps2 functions as an integral part of the LINC complex interacting with Mps3. We observed that Mps2 localizes at the end of chromosomes, Mps2-Mps3 physically interact, and expression of Mps2 C-terminal mutation (six amino acids) impairs active prophase chromosome movements [36]. This provides substantial evidence that Mps2 functions are comparable to those of a KASH-like protein within the LINC complex, connecting the Mps3/Ndj1/telomere complex to the cytoskeleton.

## Identification of Mps2 interacting proteins, candidates for novel factors participating in active movements

We propose a model in which the N terminus of Mps2 is located in the cytoplasm, and the C terminus in the intermembrane lumen (Figure 1). To test this model we performed a yeast two-hybrid screen [36] using amino acids 1–310 of Mps2 as bait to screen a prey library of yeast genomic DNA fragments [40].

We identified a number of possible interactors for Mps2 among which we highlight myosin 2 (Myo2) [36]. Myo2 is a type V myosin with well-defined domains corresponding to a cargo binding region, which possibly interacts with adaptor proteins [41]. The interaction was confirmed by co-immunoprecipitation in yeast co-expressing Myo2-HA and Mps2-Myc. Our experiments further demonstrated that: (1) Myo2 interacts with the LINC complex, (2) Mps2 is required to recruit Myo2 to the nuclear envelope during meiosis, and (3) expression of a dominant-negative allele of Myo2 (*Myo2ΔN*) delocalizes Myo2 from nuclear envelope, perturbs RPMs, and delays meiotic progression [36].

## Csm4 regulates Mps2-Myo2 RPM functions

Csm4, a tail-anchored nuclear envelope protein, localizes at telomeric ends of chromosomes in meiotic chromosome spreads, suggesting its involvement in the LINC complex. Deletion of Csm4 results in a strong impairment of RPMs [42,43]. Our data suggest that in meiosis, Csm4 acts as a regulator of Mps2 and Myo2 functions. This is supported by our findings [36]: (1) Csm4 does not influence the Mps2-Mps3 interaction and Csm4 localization at telomeres depends on Mps2. (2) Coimmunoprecipitation experiments show that Mps2 and Csm4 interact during meiosis. (3) Deletion of Csm4 or Mps2 leads to loss of Myo2-GFP association to the nuclear envelope. Together, these results suggest that Csm4 and Mps2 are both positioned in the outer nuclear membrane where they provide the link with extranuclear components associated with the LINC complex.

**Figure 2.**
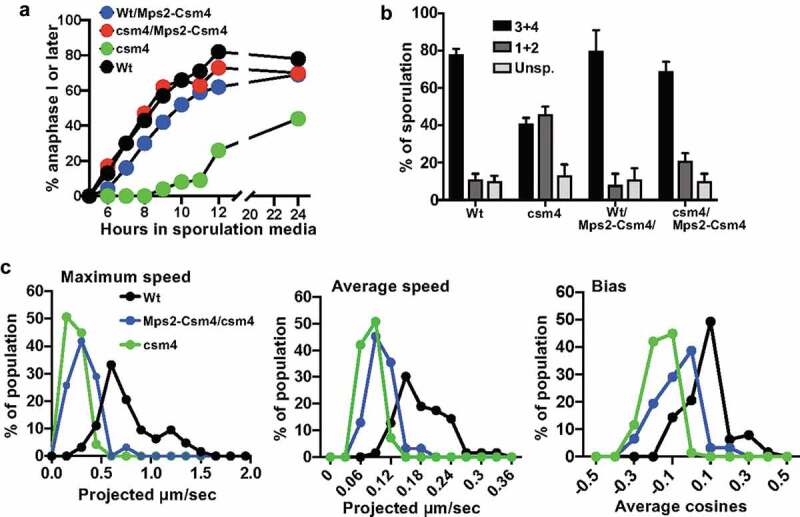
Characterization of the Mps2-Csm4 fusion protein. (a) Csm4-Mps2 fusion was generated by introducing a SmaI site immediately upstream of the MPS2 start codon to permit fusion to YFP-Csm4 after codon 130 of Csm4 thus deleting the transmembrane domain from CSM4. These constructs were expressed from the CSM4 promoter and cloned onto MCB917. Construction of csm4 strain was described in [36]. Expression of Mps2-Csm4 complements the csm4 deletion meiotic defect. Mps2-Csm4 expression in wild type has no apparent effect in meiosis, finishing with wild-type rates of sporulation. For this experiment, cells were synchronized in liquid YPA and shifted into sporulation medium (dextrose 0.05%, potassium acetate 1%, yeast extract 0.1%) at 30°C. The first meiotic division starts 5 hours following the shift into sporulation medium. Cells were stained with DAPI (DAPI 0.05% in ethanol 100%) and 200 cells per time point in each strain were scored using fluorescence microscope. (b) Sporulation rate in solid medium plates of wild type, csm4, and wild type and csm4 expressing the Mps2-Csm4 fusion. Two hundred cells were scored for sporulation per experiment per strain. The categories used: unsporulated, units containing one or two spores, and units containing three or four spores. Results show average and standard deviation obtained from at least three independent experiments. (c) Histograms display RPM activity by measures of maximum speed, average speed, and bias for paired chromosome 4 right telomeres in wild-type, *csm4*, and Mps2-Csm4 strain. In our strain background, for strains that are not delayed in meiotic progression 5-hours post-shift correlates with the peak of cells in pachytene. For *csm4*, a mutant known for prolonged delay, 8-hours post-shift roughly correlates with the peak of cells in pachytene. Diploids were grown 24 hours in 5 mL YPDA medium at 30°C. For synchronous meiosis cells were grown to 5 × 10^7^ cells/ml in YPAcetate medium for 16 hours, then shifted to 1% potassium acetate at 10^8^ cells/ml. Each profile is the result of a total of three independent experiments (250–300 cells scored per strain), where cells have 1 spot (paired homologous chromosomes). Note that the ‘bias’ measure, the average of the cosines of the angles made between successive movements, is unitless. Detailed methods regarding through-focus microscopy and semi-automatic quantification and analyses were published in [32]. Prism – GraphPad was used for generation of graphs.

Prompted by the observed interaction between Mps2 and Csm4 [36] and to further analyze the role of Mps2-Csm4 cooperation, we generated an Mps2-Csm4 fusion protein and tested its effect in wild type and csm4 mutant. In this manuscript, we show the results obtained studying sporulation, anaphase I entry, and RPMs. We fused amino acids 1–130 of Csm4 to the N-terminus of full-length Mps2 and expressed the fusion gene from the CSM4 promoter (detail in the legend of Figure 2). Expression of Mps2-Csm4 in the wild-type strain did not have any apparent effect on anaphase I entry or sporulation rate (Figure 2(a)). Importantly, Mps2-Csm4 fusion complemented the prophase delay observed in csm4 (Figure 2(a)) and suppressed the csm4 defect in sporulation rate (Figure 2(b)).

We then measured RPMs in wild type, csm4 mutant, and Mps2-Csm4 fusion strain. We acquired time-lapse images of GFP-lacI bound to lacO sequences integrated near the right telomere of paired chromosome *lV* and measured the maximum speed, average speed, and bias. Csm4 deletion mutant was used as a control for reduced RPMs. We found that RPMs are partially rescued in Mps2-Csm4 fusion strain when compared with csm4 deletion, suggesting partial complementation of the *csm4* deletion (Figure 2(c)). The most notable change in the character of telomere movements was detected by an increase in bias, which describes the ability for a spot to move away from its starting point. It is noteworthy that moderate enhancements of movement provided by the Csm4-Mps2 fusion protein have very significant effects on the meiotic outcome. While it is possible that the fusion protein represents some separation of function allele, we favor the hypothesis that the fusion protein is simply a hypomorphic allele of Csm4 that is not quite in its normal geometry and the fusion protein must compete with Mps2 for telomere association. These observations may suggest that in many circumstances very low levels of movement may be sufficient to accomplish meiosis in budding yeast. It is also possible that bias may be more critical than speed. There may be some critical event, such as an interlock, that absolutely requires movement to resolve, while other processes, such as the homology search are stimulated by movement but can often be completed, but more slowly when movement is compromised. Finally, the relatively mild rescue phenotype may be also interpreted as that the dynamic interactions between Mps2 and Csm4 play an important role in triggering/regulating RPMs.

Overall, our work helps build a model for the engine generating RPMs in *S. cerevisiae*. We highlight our recent work [36] showing that Mps2 is part of the internuclear membrane complex connecting telomeres with cytoskeleton components; we identify Myo2 motor protein, which interacts with Mps2 connecting telomeres to the actin cytoskeleton; and show that Csm4 is a regulator of the Mps2-Myo2 interaction and function. Additionally, in this manuscript, we show previously unpublished results pertaining to the functional interaction of Mps2 and Csm4. We show that an Mps2-Csm4 fusion protein can complement deficient sporulation and anaphase I entrance of a csm4 deletion strain, and partially complement RPM functions.
